# Supplementary material for: Structural basis for GPR40 allosteric agonism and incretin stimulation
Source: Nat Commun. 2018 Apr 25;9:1645. doi: 10.1038/s41467-017-01240-w (PMC5917010; doi:10.1038/s41467-017-01240-w)
Supplement: Supplementary file 1 — Supplementary Information [file 41467_2017_1240_MOESM1_ESM.pdf]

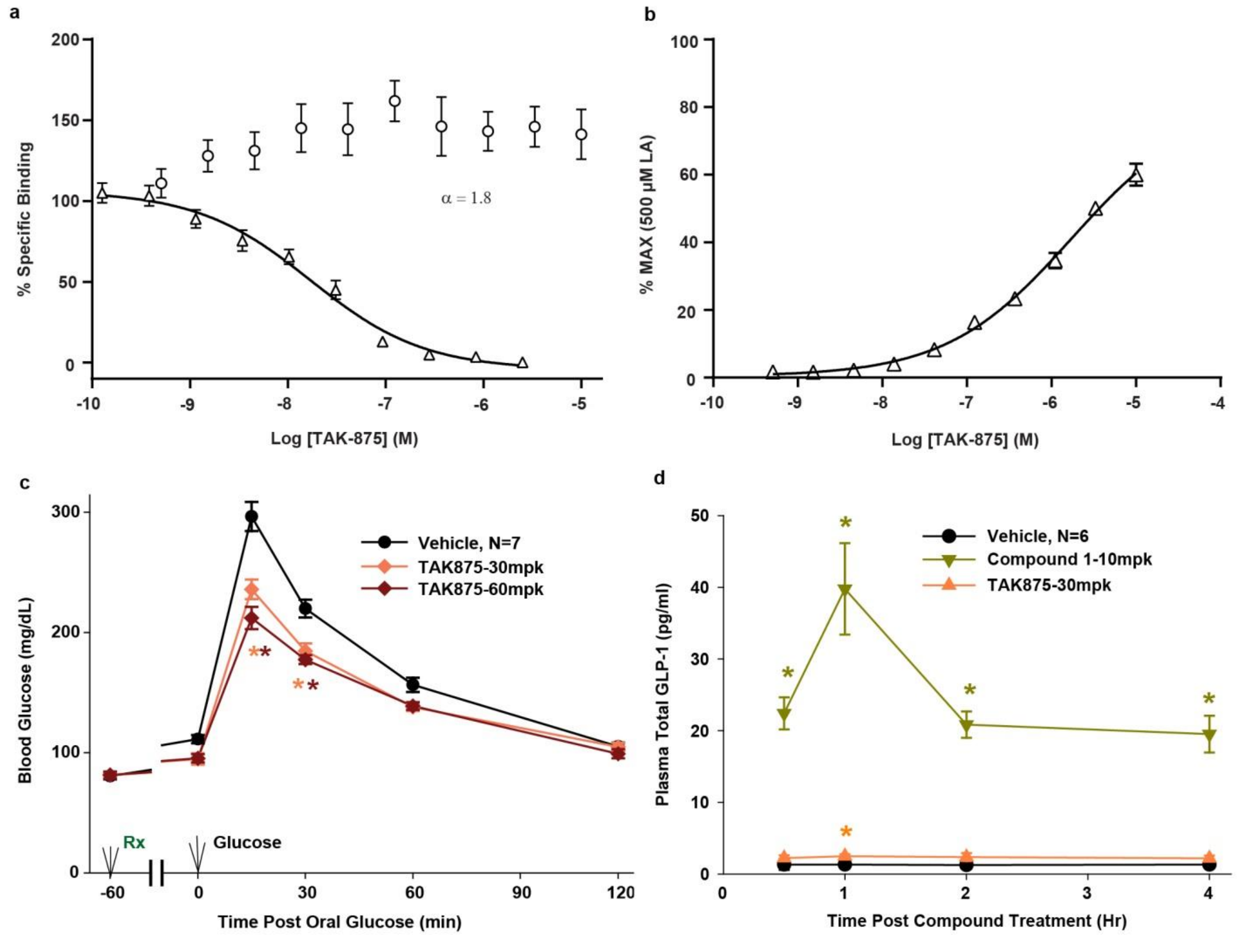

Supplementary Figure 1. Pharmacology of TAK-875. **a**, Competitive radioligand binding for TAK-875. GPR40 membranes (prepared from stably transfected cells) were incubated with either [^{3}H]-TAK-875 (Δ) or [^{3}H]-AM-1638 (○) in the presence of unlabeled TAK-875, as described in the Methods section. Percent specific binding (y-axis) was plotted against the log concentration of TAK-875 (x-axis) and fit with a 4 parameter nonlinear logistic curve function with variable slope (GraphPad Prism, v7.00). The K_i for TAK-875 in [^{3}H]-TAK-875 binding was 17 nM. An inhibition curve for [^{3}H]-AM-1638 binding was not drawn since the addition of TAK-875 resulted in enhanced binding activity. Data points represent the mean of at least three independent experiments with error bars indicating s.e.m. The affinity modulation factor (α) was calculated using the allosteric modulator titration equation within GraphPad Prism. **b**, Ca^{2+} mobilization assay (FLIPR®) using stably transfected cells. GPR40- $G\alpha_q$ signaling by TAK-875 (Δ). TAK-875 acts as a partial agonist relative to 500 µM of the natural ligand, linoleic acid. (n=4; EC_{50} ~1.71μM; Top 81.67%; error bars represent s.e.m.) **c,** OGTT in wild type C57BL/6 mice. Decreases in glucose are seen after treatment with TAK-875 at 30 and 60 mg kg^{-1}. **d**, Incretin assay in wild type C57BL/6 mice. Similar to vehicle, TAK-875 has no effect on plasma total GLP-1 post an oral treatment at 30 mg/kg. Oral treatment of Compound **1** at 10 mg/kg sustains an elevated level of plasma total GLP-1 in the 4-hour window.

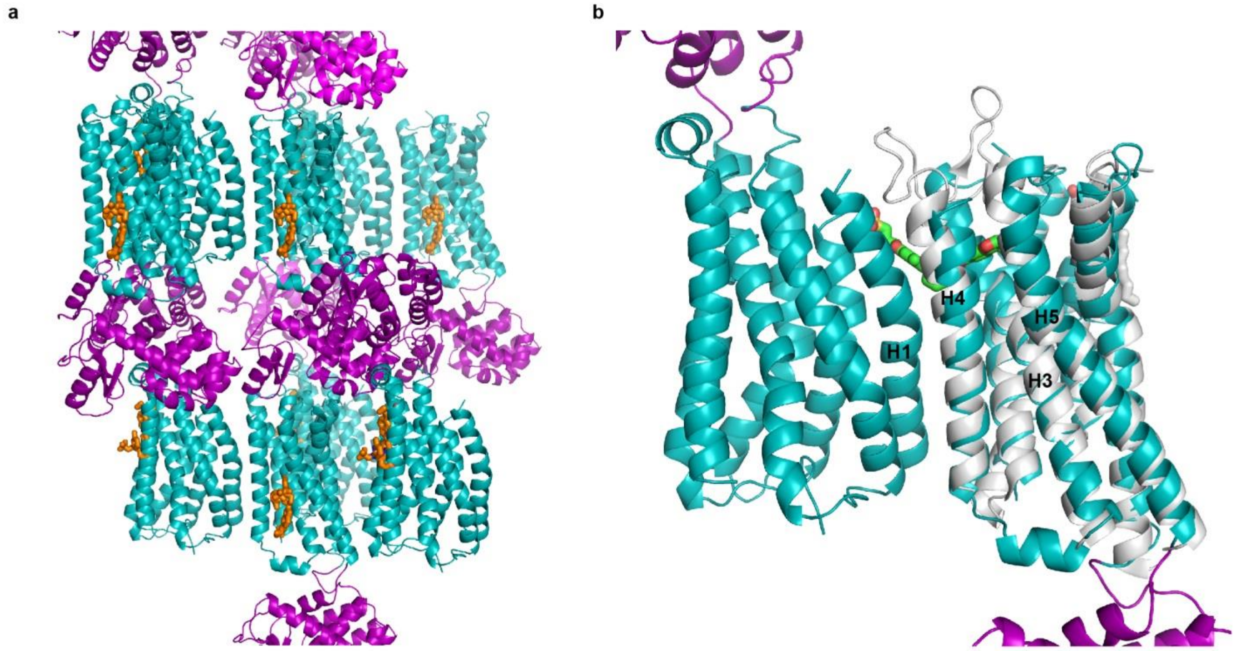

Supplementary Figure 2. a, Crystal lattice of the GPR40-compound 1 structure. The GPR40 receptor is in cyan; T4L is in magenta; Compound **1** is in orange as sticks. The binding site for compound **1** is open to solvent and not affected by crystal packing contact. **b, Superposition of 4PHU in the crystal packing interface of GPR40-compound 1.** GPR40 from 4PHU is in white; TAK-875 is in green; the GPR40-compound **1** structure is in cyan; compound **1** is removed for clarity. The superposition shows that the A1 site in the GPR40-compound **1** structure is perturbed by crystal packing contacts with a neighboring GPR40 molecule.

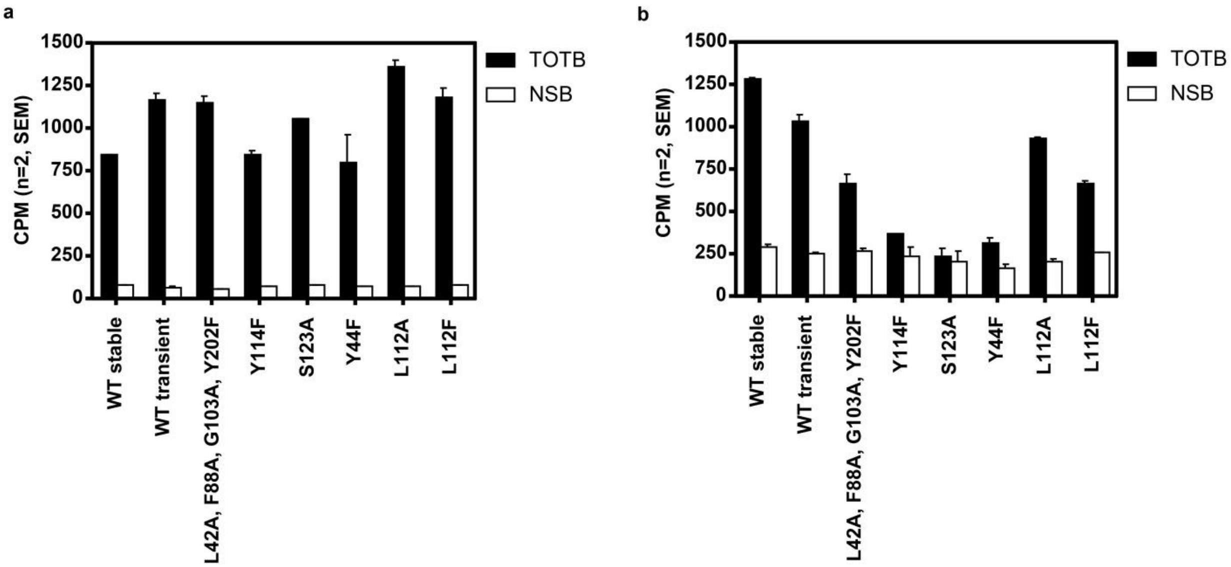

c

	TAK-875		AM 1638	
**GPR40 Construct**	**Kd, nM**	**Bmax, pmol/mg**	**Kd, nM**	**Bmax, pmol/mg**
WT Stable	3.1	31	3.8	13
WT Transient	2.5	38	2.2	11
L42A, F88A, G103A, Y20F	4.1	52	29	28
Y114F	2.9	30	3.2	3.1
S123A	4.4	49	No Fit	No Fit
Y44F	3.3	33	No Fit	No Fit
L112A	3.7	55	1.8	5.5
L112F	3.2	44	1.9	5.7

Supplementary Figure 3. Radioligand Binding for GPR40 Control and Mutant Constructs. Radioligand binding experiments were conducted as described in the Methods section. Total binding (solid bars) and nonspecific binding (open bars) for membranes prepared from cells transiently transfected with no GPR40 mutation (Control), four stabilizing mutations (L42A, F88A, G103A, Y202F), single point A2 site mutations (Y114F; S123A; Y44F) and single point ICL2 mutations involved in $G\alpha_s$ coupling (L112A; L112F). Data points represent the mean of two replicates from a single experiment with error bars indicating s.e.m. **a**, [^{3}H]-TAK-875 binding (2.5 μg membrane for each) indicates that A1 site binding was preserved across the wild type and mutants, while **b**, [^{3}H]-AM-1638 binding (2.5 μg membrane for each) demonstrates the significant loss of A2 site activity for the single point A2 site mutations and reduced relative binding for the construct with the four stabilizing mutations and L112 mutations. **c**, Saturation binding analysis for [^{3}H]-TAK-875 and [^{3}H]-AM-1638 using membranes from control and mutant GPR40 constructs; shown are results from a representative experiment.

a

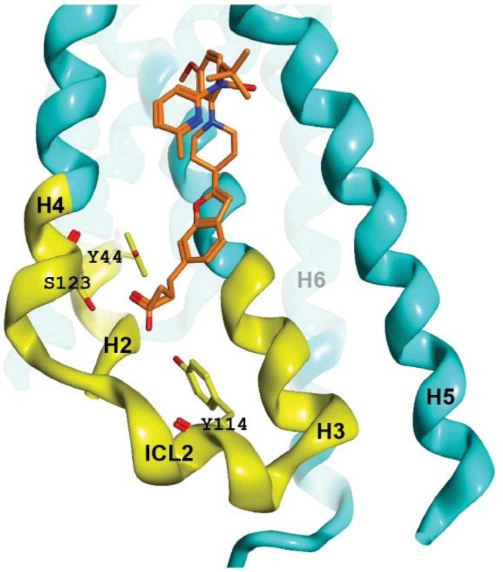

b

	H2							
	2.37	2.38	2.39	2.40	2.41	2.42	2.43	2.44
**FFAR1**	T	P	S	L	V	Y44	A	L
**FFAR2**	A	P	V	H	I	L47	L	L
**FFAR3**	V	A	V	D	V	L53	L	L
**FFAR4**	G	A	T	A	C	L77	V	L
**LPAR1**	F	P	I	Y	Y	L87	M	A
**LPAR2**	Q	P	I	Y	Y	L70	L	G
**LPAR3**	F	P	F	Y	Y	L68	L	A
**LPAR4**	S	E	T	A	I	F77	I	T
**LPAR5**	S	V	V	S	V	Y60	M	C
**LPAR6**	N	E	T	T	T	Y55	M	I
**S1P1R**	R	P	M	Y	Y	F83	I	G
**S1P2R**	S	A	M	Y	L	F71	L	G
**S1P3R**	N	R	M	Y	F	F77	I	G
**S1P4R**	R	W	V	Y	Y	C84	L	V
**S1P5R**	A	P	M	F	L	L74	L	G
**EP1R**	A	T	F	L	L	F76	V	A
**EP2R**	S	L	F	H	V	L70	V	T
**EP3R**	K	S	F	L	L	C91	I	G
**EP4R**	T	T	F	Y	T	L57	V	C

c

	H3								ICL2											H4					
	3.49	3.50	3.51	3.52	3.53	3.54	3.55	3.56												4.39	4.40	4.41	4.42	4.43	4.44
**FFAR1**	G	R104	Y	L	G	A	A	F	-	-	P	L	G	Y114	Q	A	F	R	R	P	C	Y	S123	W	G
**FFAR2**	E	R107	Y	L	G	V	A	F	-	-	P	V	Q	Y117	K	L	S	R	R	P	L	Y	G126	V	I
**FFAR3**	E	R113	F	L	S	V	A	H	-	-	P	L	W	Y123	K	T	R	P	R	L	G	Q	A132	G	L
**FFAR4**	E	R136	M	V	C	I	V	H	-	-	L	Q	R	G146	V	R	G	P	G	R	R	A	R155	A	V
**LPAR1**	E	R146	H	I	T	V	F	R	-	-	M	Q	L	H156	T	R	M	S	-	N	R	R	V164	V	V
**LPAR2**	E	R129	H	R	S	V	M	A	-	-	V	Q	L	H139	S	R	L	P	-	R	G	R	V147	V	M
**LPAR3**	E	R127	H	M	S	I	M	R	-	-	M	R	V	H137	S	N	L	T	-	K	K	R	V145	T	L
**LPAR4**	D	R136	F	L	A	I	V	Y	-	-	P	F	R	S146	R	T	I	R	T	R	R	N	S155	A	I
**LPAR5**	D	R119	Y	A	A	I	V	H	-	-	P	L	R	L129	R	H	L	R	R	P	R	V	A138	R	L
**LPAR6**	D	R114	F	L	A	I	V	Y	-	-	P	F	K	S124	K	T	L	R	T	K	R	N	A133	K	I
**S1P1R**	E	R142	Y	I	T	M	L	K	-	-	M	K	L	H152	N	G	S	N	-	N	F	R	L160	F	L
**S1P2R**	E	R130	H	V	A	I	A	K	-	-	V	K	L	Y140	G	S	D	K	-	S	C	R	M148	L	L
**S1P3R**	E	R136	H	L	T	M	I	K	-	-	M	R	P	Y146	D	A	N	K	-	R	H	R	V154	F	L
**S1P4R**	E	R143	F	A	T	M	V	R	-	-	P	V	A	E153	S	G	A	T	K	T	S	R	V162	Y	G
**S1P5R**	E	R133	S	L	T	M	A	R	R	G	P	A	P	V145	S	S	-	-	-	R	G	R	T151	L	A
**EP1R**	E	R135	C	V	G	V	T	R	-	-	P	L	L	H145	A	A	R	V	S	V	A	R	A154	R	L
**EP2R**	E	R134	Y	L	S	I	G	H	-	-	P	Y	F	Y144	Q	R	R	V	S	R	S	G	G153	L	A
**EP3R**	E	R155	A	L	A	I	R	A	-	-	P	H	W	Y165	A	S	H	M	K	T	R	A	T174	R	A
**EP4R**	E	R117	Y	L	A	I	N	H	-	-	A	Y	F	Y127	S	H	Y	V	D	K	R	L	A136	G	L

Supplementary Figure 4. Sequence alignment of four classes of human lipid receptors: free fatty acid receptors (FFAR), sphingosine-1-phosphate receptors (S1PR), lysophosphatidic acid receptor (LPAR), prostaglandin E receptors (EPR). a, The A2 binding pocket. Receptor is colored in cyan, and compound **1** in orange. Ribbon represents the transmembrane helices. Y44$^{2.42}$, Y114^{ICL2}, S123$^{4.42}$ are drawn as sticks. Focus of the alignment is colored in yellow. **b, c,** Alignment covers a part of H2, part of H3, the entire ICL2, and part of H4 as defined by the yellow area in (**a**).

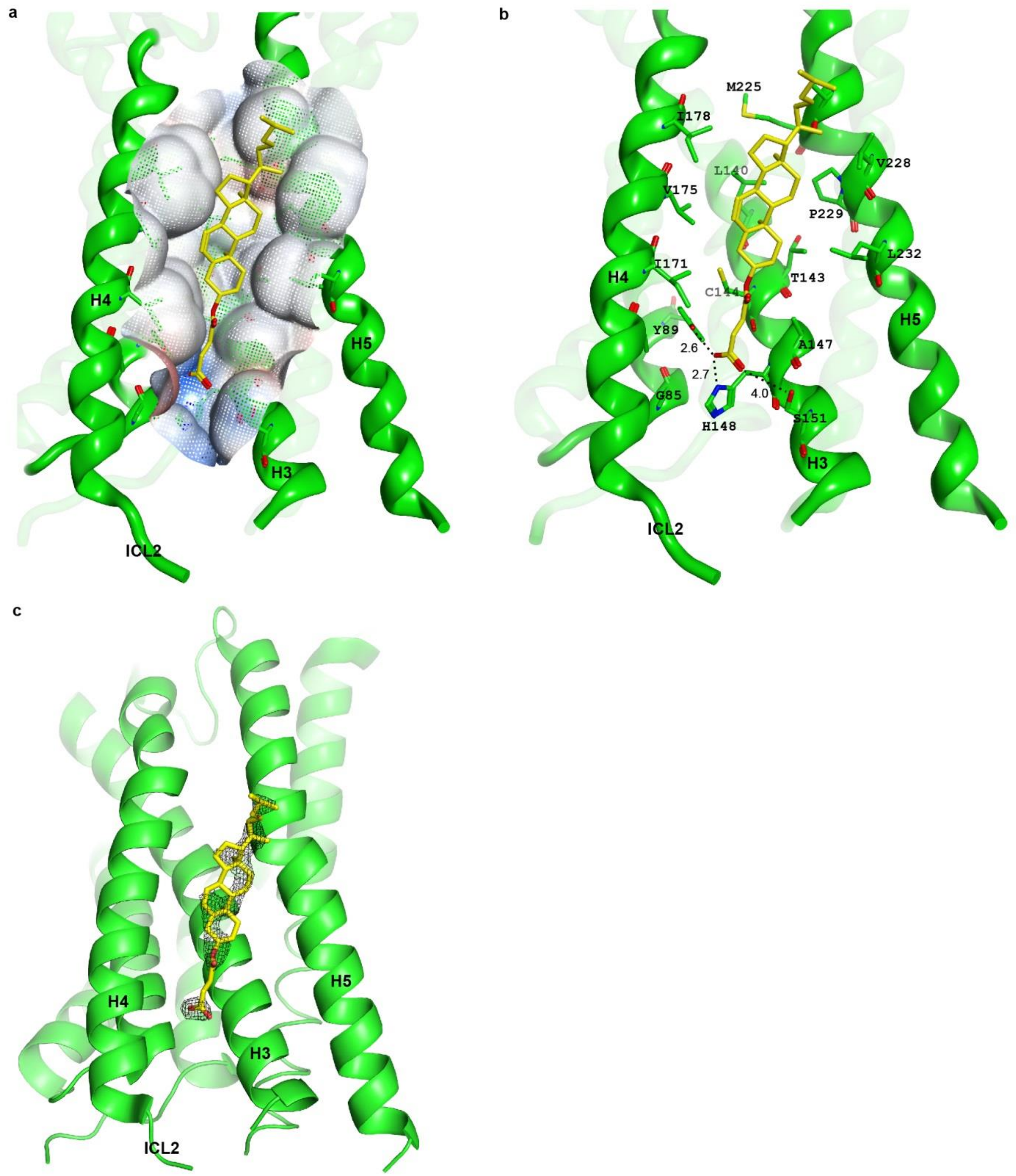

Supplementary Figure 5. Binding interaction of cholesteryl hemisuccinate (CHS) in P2Y$_1$R. a. In silver mesh is an "interaction surface" of the region around the CHS molecule, which indicates that the ligand is within optimal van der Waals (vdW) contact distance when touching the surface. P2Y$_1$R is colored in green and CHS in yellow with oxygen atoms in red. Ribbon represents the transmembrane helices. **b**, A more detailed view of the binding site. Hydrogen bonds are represented as dotted lines. Distances shown are between oxygen and oxygen (nitrogen on the histidine). **c**, The F_o-F_c omit map of CHS was calculated in its absence and are shown as black mesh and contoured at 3.0 σ. Transmembrane helices H3, H4, H5 and ICL2 are labeled.

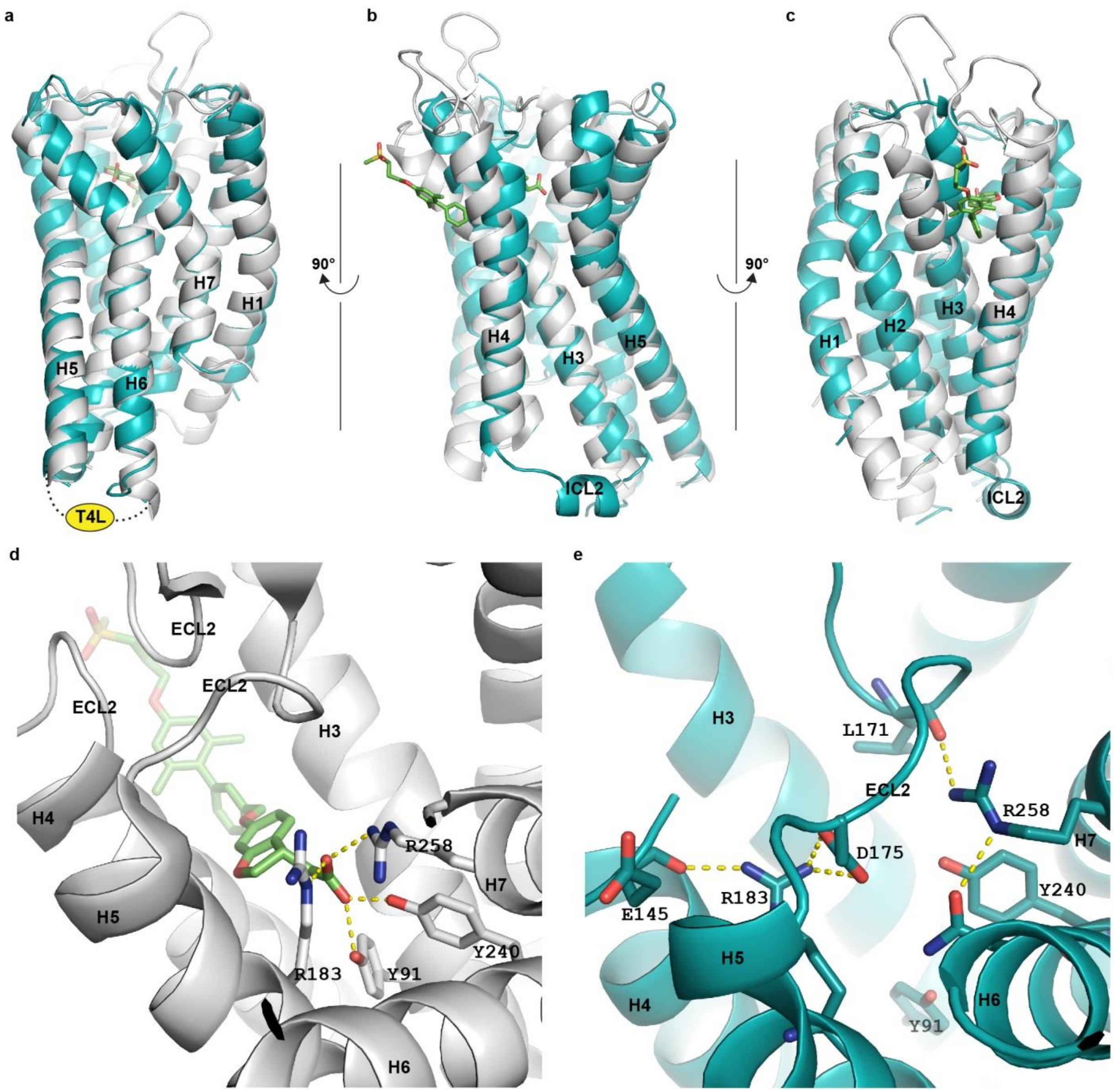

Supplementary Figure 6. Structural superposition of GPR40-compound 1 with GPR40-TAK-875 (PDB 4PHU). a-c, 4PHU is shown as GPR40 in white ribbon and TAK-875 in green stick. GPR40-compound **1** complex is shown in cyan ribbon with compound **1** removed for clarity. Transmembrane helices and intracellular loop 2 (ICL2) are labeled. **a**, T4L fusion was inserted between Helix 5 and 6 in the crystallization construct. **b**, viewed parallel to the membrane with (**a**) and (**c**) as opposing 90° vertical rotation from (**b**). Cα RMSD=1.05 Å. **d**, Site A1 in GPR40-TAK-875. **e**, Site A1 in GPR40-compound **1**.

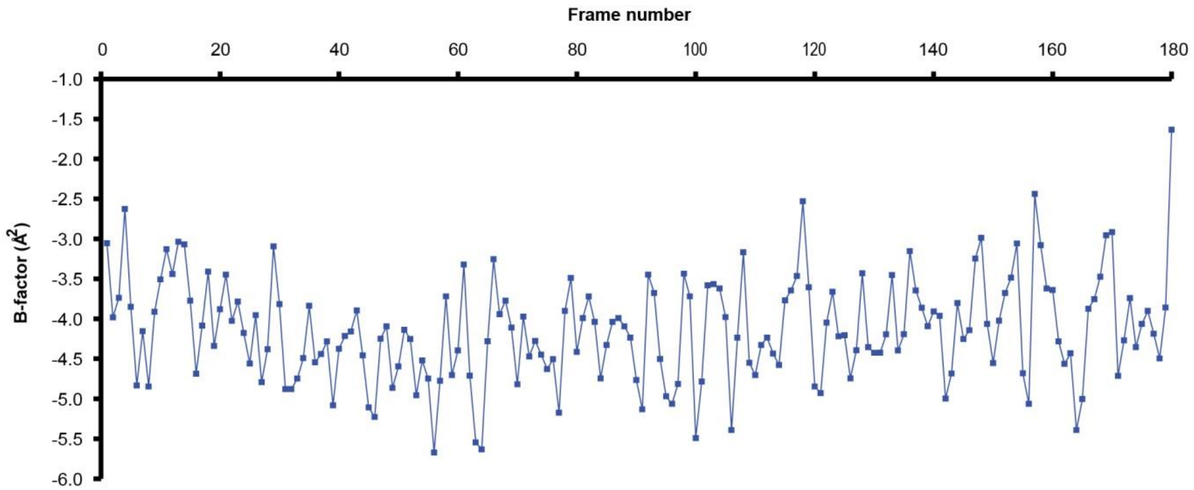

Supplementary Figure 7. Graph showing the B-factor vs. diffraction frame number as obtained from scaling the data, indicating negligible crystal decay during data collection.

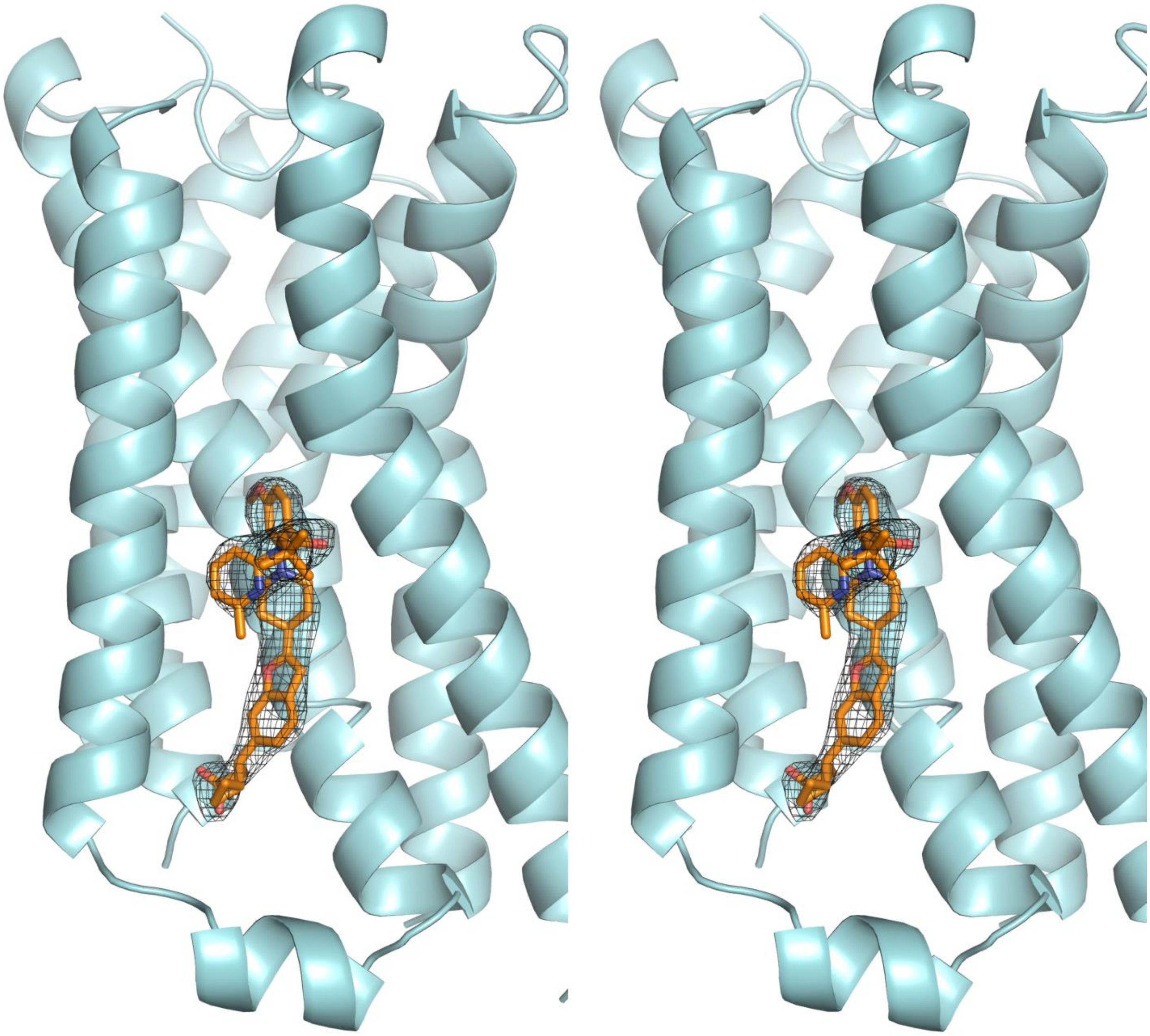

Supplementary Figure 8. Stereo image of the GPR40-compound **1** complex shown as ribbon representation. Compound **1** is shown as stick in orange. The F_o-F_c omit map of compound **1** was calculated in its absence and is shown as black mesh and contoured at 3.0 σ. Figure was prepared using PyMOL (Schrödinger, New York).

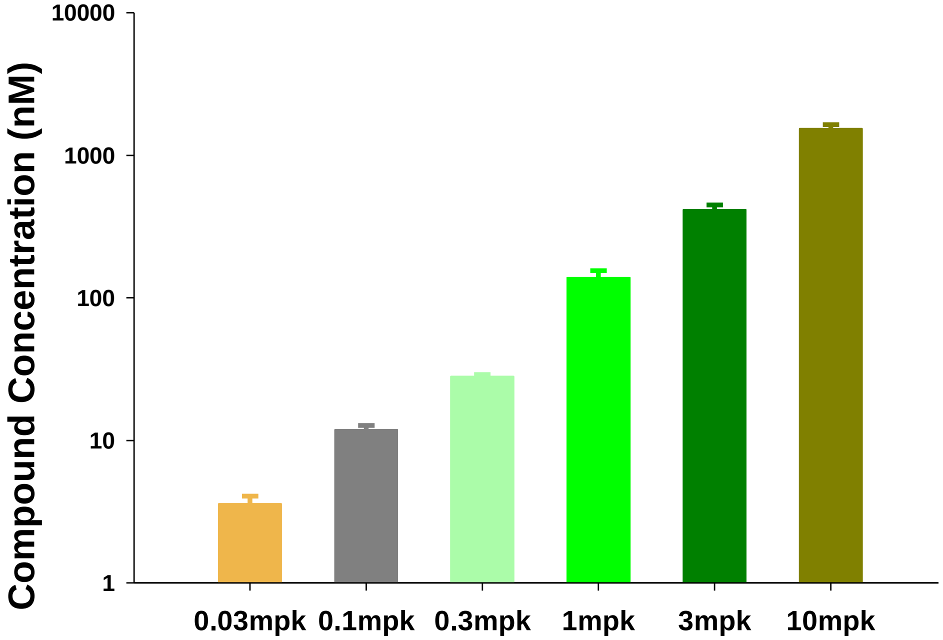

Supplementary Figure 9. Drug exposure in plasma after oral dose of compound 1 in wild type C57BL/6 mice at various doses.

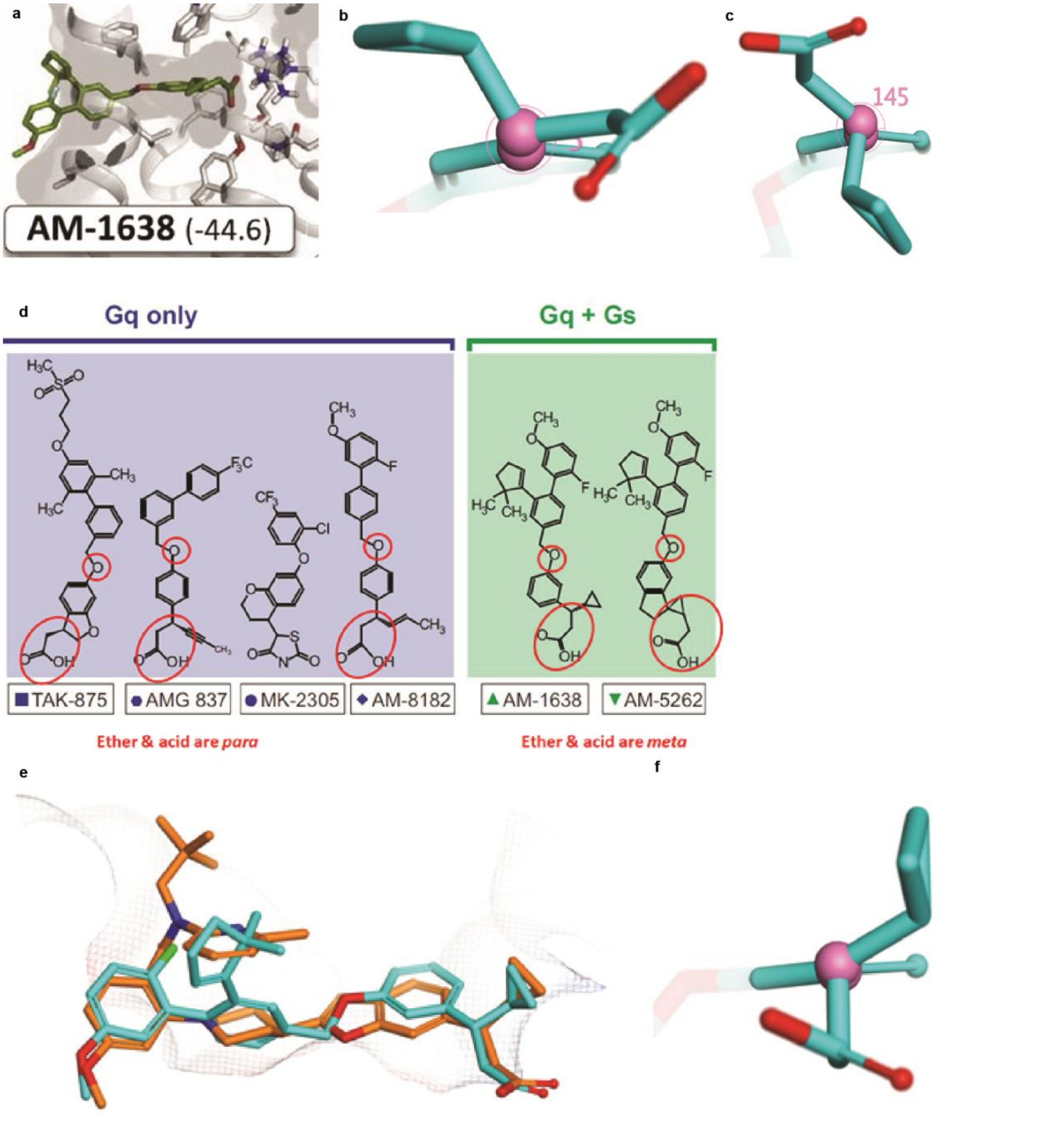

Supplementary Figure 10. a, Figure adapted from Hauge *et al.*[1] showing their docked conformation of AM-1638 in site A1. **b,** A close-up view of the docked pose in (a), showing the highly strained conformation required for binding in site A1, with the carboxylic acid and cyclopropane on the same side of the phenyl ring. **c,** A preferred conformation of AM-1638, with the carboxylic acid and cyclopropane on opposing sides of the phenyl ring. **d,** Figure adapted from Hauge *et al.*[1] Annotation emphasizes the possible structural determinant of site A1 binders and A2 binders. **e,** AM-1638 modeled in Site A2, superposed with the crystal structure of Compound **1** and **f,** illustrating that the aforementioned torsion is in a well-tolerated, low energy conformation.

	TAK-875 (A1)	LY2881835 (A1)	AM1638 (A2)	Compound 1 (A2)
Human GPR40 Binding to A1 Ki, nM	7.7	4.7	>6910	>5590
Human GPR40 Binding to A2 Ki, nM	>7050	>3720	1.4	0.9
Human GPR40 Calcium EC50, nM (Emax, %)	159 (67)	9.1 (62)	1.7 (98)	1.8 (110)
Human GPR40 b-arrestin EC50, nM (Emax, %)	11.5 (119)	8.7 (137)	16 (153)	1.9 (172)
Mouse GPR40 b-arrestin EC50, nM (Emax, %)	8.6 (117)	0.8 (133)	22.7 (112)	1.6 (146)
Rat GPR40 b-arrestin EC50, nM (Emax, %)	36	2.0 (124)	105 (113)	2.1 (163)
Human GPR40 IP1 EC50, nM (Emax, %)	0.35 (145)	1.24 (162)	0.25 (197)	<0.05 (484)
Human GPR120 Binding to Ki, nM	>100000	>100000	ND	>100000
Human GPR120 Calcium EC50, nM (Emax, %)	>50000	>50000	2870 (63)	>50000
Human GPR120 b-arrestin EC50, nM (Emax, %)	>100000	>100000	ND	>100000
Mouse GPR120 b-arrestin EC50, nM (Emax, %)	>50000	>50000	ND	>50000
human PPARalpha binding, nM	>10000	>10000	3170	>10000
human PPARdelta binding, nM	>10000	>10000	>10000	>10000
human PPARgama binding, nM	>10000	>10000	>10000	>10000

Supplementary Table 1. Representative GPR40 site A1 and A2 binders and their activities.

	Compound 1
hPPARalpha binding Ki (nM)	>10000
hPPARdelta binding Rel IC50 (nM)	>10000
hPPARgamma binding Ki (nM)	>10000
hRXRalpha binding Ki (nM)	>10000
hRORalpha binding Ki (nM)	>20000
hRORbeta binding Ki (nM)	>13000
hRORgamma binding Ki (nM)	>20000
hERalpha binding Ki (nM)	>11000
hGPR120 beta-Arrestin Rel EC50 (nM)	>100000
hGPR120 FLIPR Rel EC50 (nM)	>50000
hGPR120 binding Ki (nM)	>100000
hGLUCR binding Ki (nM)	>10000
hM1 binding Rel IC50 (nM)	>10000
hD1 cAMP Rel EC50 (nM)	>80000
hNav1.7 binding Rel IC50 (nM)	>30000
hCDK1 binding IC50 (nM)	>20000
hCDK2 binding IC50 (uM)	>20000

Supplementary Table 2. Compound 1 selectivity panel.

Dog Hepatocyte Clearance ABT		Human Hepatocyte Clearance ABT		Mouse Hepatocyte Clearance ABT		Rat Hepatocyte Clearance ABT		Dog Hepatocyte Clearance no ABT		Human Hepatocyte Clearance no ABT		Mouse Hepatocyte Clearance no ABT		Rat Hepatocyte Clearance no ABT	
Intrinsic Clearance (ul/min/milcells)	% Recovery	Intrinsic Clearance (ul/min/milcells)	% Recovery	Intrinsic Clearance (ul/min/milcells)	% Recovery	Intrinsic Clearance (ul/min/milcells)	% Recovery	Intrinsic Clearance (ul/min/milcells)	% Recovery	Intrinsic Clearance (ul/min/milcells)	% Recovery	Intrinsic Clearance (ul/min/milcells)	% Recovery	Intrinsic Clearance (ul/min/milcells)	% Recovery
19.3	92.8	<1.8	92.8	12.4	92.8	3	92.8	31.4	103	8.8	103	9.2	103	12.2	103

Supplementary Table 3. Hepatic clearance of compound 1 in various species.

Supplementary Discussion:

SAR, *in vitro* data, selectivity, metabolism and PK

Identification and optimization of our initial Lead series focused on a careful examination of the three key areas of the pharmacophore: *the acidic head group, the center linker and the hydrophobic tail.* Inspired by subsequent reports on GPR40 agonists, we undertook a focused structure–activity relationship (SAR) around these three key areas. Our initial efforts focused on improving the *in vitro* potency and pharmacokinetic (PK) properties, and eliminating the risk of PPAR activity. The carboxylic acid function is typically attached by a 2 carbon atom linker to the meta position of a phenyl ring or to a heterobicyclic ring such as a benzofurane. A broad range of substituted propionic acid analogues were made and examined for their *in vitro* and *in vivo* properties. Supplementary Table 1 shows a comparison of the binding and functional data between a typical allosteric A1 that binds selectively to the A1 site such as TAK-875 and LY2881835 and an allosteric A2 that binds selectively to the A2 site such as compound **1**.

Since ligands to PPAR α, δ and γ are known to have therapeutic benefits but with significant risks, part of our SAR strategy was to eliminate all PPAR activity from our GPR40 agonists. Compound **1** was tested in all PPAR α,δ,γ binding and functional assays at multiple concentrations. The data demonstrated lack of significant PPAR binding or functional activities. Furthermore, compound **1** was highly selective when tested in selectivity panels against representative of GPCRs (including the most related targets such as GPR120), NHRs and Kinases. Supplementary Table 2 summarizes the data from some of the key representative selectivity targets that were identified as a risk and we drove the SAR away from them. Compound **1** showed to be highly selective against these targets.

We found that the hepatocyte metabolism was the rate-limiting step in the hepatic clearance of this class of molecules. Therefore, hepatocyte clearance measurements were a paramount consideration during the optimization and selection of compounds. Supplementary Table 3 shows the hepatocyte intrinsic clearance across species for compound **1.**

In vivo metabolism of this class of compounds was investigated in plasma and urine. The parent drug was shown to be the major circulating component observed in plasma and only one plasma metabolite was observed (parent + glucuronide). The parent drug was also the major component observed in the urine sample. All metabolites in urine and bile were minor based on MS peak intensities. This result was also consistent with the hepatocyte incubation results.

Oral glucose tolerance test (OGTT) in wild type C57BL/6 mice showed dose dependent decreases in glucose excursion after treatment with various doses of compound **1.** The pharmacokinetics in in mouse was dose proportional; the drug exposures in plasma increased with increasing dose (Supplementary Fig. 9).

Docking comparison of AM-1638 in site A1 vs. A2

We modeled AM-1638 in sites A1 and A2. We were able to reproduce the same binding mode of AM-1638 in A1 as reported by Hauge *et al*.[1] (Supplementary Fig. 10a), in which the carboxylic acid and cyclopropane are on the same side of the proximal phenyl ring (Supplementary Fig. 10b), a strained conformation in which the two ß-position carbons sterically penetrate through the vdW radii of phenyl ring carbons. In the more energetically stable conformations of AM-1638, the carboxylic acid and the cyclopropane reside on opposing sides of the phenyl ring, as shown in Supplementary Fig. 10c. When comparing the Gq only ligands with the Gq + Gs ligands, the methoxy linkage and carboxylic acid head groups are *para* substituted in the Gq only ligands and *meta* substituted in the Gq + Gs ligands (Supplementary Fig. 10d). With the *meta* substitution pattern, it appears that the acid can only get to the requisite Arg 183 and Arg 258 with a strained ligand conformation as indicated in Supplementary Fig. 10a-b. When AM-1638 is modeled in site A2, there is a good spatial overlap with the experimental binding mode of compound **1** (Supplementary Fig. 10e) and the torsion of bond from the phenyl to the α carbon is energetically favorable (Supplementary Fig. 10f).

Supplementary Method: Synthesis of Compound 1

Preparation 1

2,2-dimethyl-N-(6-methyl-2-pyridyl)propanamide

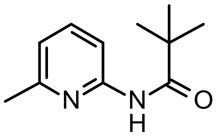

The solution of 6-methylpyridin-2-amine (51.2 g, 473.46 mmoles) in DCM (300 mL) was added N-ethyl-N-isopropylpropan-2-amine (108 mL, 615.49 mmoles), and then cooled to 0°C. The solution of pivaloyl chloride (64 mL, 520.80 mmoles) in DCM (200 mL) was added dropwise. After addition, the mixture was stirred at room temperature for 3 hr. The mixture was washed with water, saturated Na_2CO_3 solution, brine, dried over Na_2SO_4, and concentrated under reduced pressure to get N-(6-methylpyridin-2-yl)pivalamide (84 g; 436.91 mmoles; 92% yield) as pale yellow solid.

LC-MS (UV 214): 193.3 (M+H)+ (100 % purity). 1H NMR (400 MHz, CDCl3) δ 8.82 (s, 1H), 8.17 (d, J = 8.4 Hz, 1H), 7.69 (t, J = 8.0 Hz, 1H), 6.93 (d, J = 7.6 Hz, 1H), 2.52 (s, 3H), 1.35 (s, 9H).

Preparation 2

N-(2,2-dimethylpropyl)-6-methyl-pyridin-2-amine

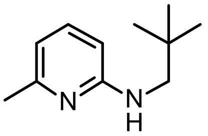

The suspension of $LiAlH_4$ in THF (312 mL, 312 mmoles) was cooled to 0°C. The solution of N-(6-methylpyridin-2-yl)pivalamide (20 g, 104.03 mmoles) in THF (300 mL) was added dropwise. After addition, the mixture was heated at 60°C for 2 hr, and then cooled to 0°C. Water (12 mL), NaOH solution (4 mol/L, 12 mL), and water (36 mL) was with sequential added. White precipitate was removed by filtration. The filtrate was concentrated under reduced pressure to get 6-methyl-N-neopentylpyridin-2-amine (17.6 g, 95.76 mmoles; 92 % yield) as yellow oil. Results: LC-MS (UV 254): 179.1 (M+H)+ (97 % purity). 1H NMR (400 MHz, CDCl3) δ 7.32 (m, 1H), 6.42 (d, J = 7.2 Hz, 1H), 6.21 (d, J = 8.0 Hz, 1H), 4.58 (s, 1H), 2.99 (d, 2H), 2.36 (s, 3H), 0.99 (s, 9H).2.73-2.71 (m, 1H), 2.44-2.43 (m, 1H), 1.83-1.80 (m, 1H), 0.86 (s, 9H), 0.11 (d, J= 8.0 Hz, 6H).

Preparation 3

2-fluoro-4-methoxy-benzoyl chloridepropan-1-one

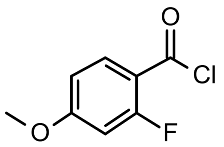

The solution of 2-fluoro-4-methoxybenzoic acid (20 g, 117.55 mmoles) in DCM (200 mL) was added DMF (0.5 mL, 6.46 mmoles). Oxalyl dichloride (12.02 mL, 141.06 mmoles) was added. The mixture was heated at 40°C for 3 hr. The mixture was concentrated under reduced pressure to get 2-fluoro-4-methoxybenzoyl chloride (22 g; 116.66 mmoles, 99 % yield) as yellow oil, which was used in next step directly.

Preparation 4

N-(2,2-dimethylpropyl)-2-fluoro-4-methoxy-N-(6-methyl-2-pyridyl)benzamide

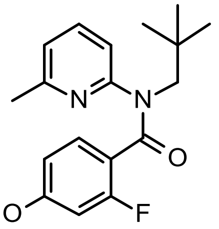

The solution of 6-methyl-N-neopentylpyridin-2-amine (30.00 g, 168.28 mmoles) in DCM (200 mL) was added N-ethyl-N-isopropylpropan-2-amine (83 mL), and then the solution of 2-fluoro-4-methoxybenzoyl chloride (38.00 g, 201.50 mmoles) in DCM (60 mL) was added dropwise. The mixture was stirred at room temperature for 3 hr. Then the mixture was washed with water, brine, dried over Na_2SO_4, and concentrated under reduced pressure. The residue was purified by chromatography on silica gel (petroleum ether / EtOAc) to get 2-fluoro-4-methoxy-N-(6-methylpyridin-2-yl)-N-neopentylbenzamide (18 g, 52.85 mmoles, 31 % yield) as white solid. Two batches of 2-fluoro-4-methoxy-N-(6-methylpyridin-2-yl)-N-neopentylbenzamide was combined, and purified further by chromatography on silica gel (petroleum ether / EtOAc) to get 2-fluoro-4-methoxy-N-(6-methylpyridin-2-yl)-N-neopentylbenzamide (26 g, 75.47 mmoles, 25 % yield) as white solid . Results: LC-MS (UV 254): 331.1 (M+H)+ (97.6 % purity);

1H NMR (400 MHz, CD3OD) δ 7.46 (t, J = 8.0 Hz, 1H), 7.13 (t, J = 8.4 Hz, 1H), 7.02 (d, J = 7.6 Hz, 1H), 6.82 (d, J = 8.0 Hz, 1H), 6.64 (dd, J = 8.4 and 2.4 Hz, 1H), 6.54 (dd, J = 12.0 and 2.4 Hz, 1H), 4.09 (s, 2H), 3.76 (s, 3H), 2.45 (s, 3H), 0.88 (s, 9H).

Preparation 5

N-(2,2-dimethylpropyl)-2-[4-(hydroxymethyl)-1-piperidyl]-4-methoxy-N-(6-methyl-2-pyridyl)benzamide

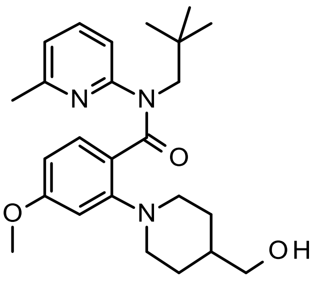

The mixture of 2-fluoro-4-methoxy-N-(6-methylpyridin-2-yl)-N-neopentylbenzamide (5.00 g, 11.93 mmoles) and piperidin-4-ylmethanol (6.97 g, 60.53 mmoles) was heated at 140°C for 6 hr, cooled to room temperature, and then partitioned between EtOAc (100 mL) and water (100 mL). The organic layer was separated, washed with brine, dried over Na_2SO_4, and concentrated under reduced pressure. The residue was purified by chromatography on silica gel (EtOAc / petroleum ether) to 2-(4-(hydroxymethyl)piperidin-1-yl)-4-methoxy-N-(6-methylpyridin-2-yl)-N-neopentylbenzamide (4 g, 9.31 mmoles, 61 % yield) as white solid. Two batches of 2-(4-(hydroxymethyl)piperidin-1-yl)-4-methoxy-N-(6-methylpyridin-2-yl)-N-neopentylbenzamide was combined, and purified further by chromatography on silica gel (petroleum ether / EtOAc) to get 2-(4-(hydroxymethyl)piperidin-1-yl)-4-methoxy-N-(6-methylpyridin-2-yl)-N-neopentylbenzamide (4.86 g, 11.42 mmoles, 47 % yield) as white solid . Results: LC-MS (UV 254): 426.2 (M+H)+ (99 % purity). 1H NMR (400 MHz, CD3OD) δ 7.22-7.28 (m, 2H), 6.88 (d, J = 7.2 Hz, 1H), 6.57 (dd, J = 8.4 and 2.4 Hz, 1H), 6.50 (m, 1H), 6.28 (s, 1H), 4.11-4.28 (m, 2H), 3.75 (s, 3H), 3.46 (d, 2H), 3.37 (m, 1H), 2.62 (m, 2H), 2.45 (m, 4H), 1.67 (m, 2H), 1.50 (m, 2H), 1.21 (m, 1H), 0.84 (s, 9H).

Preparation 6

N-(2,2-dimethylpropyl)-2-(4-formyl-1-piperidyl)-4-methoxy-N-(6-methyl-2-pyridyl)benzamide

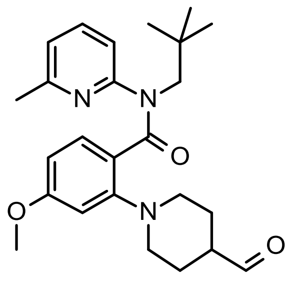

Add desmartin (0.400 g, 0.9 mmol) to N-(2,2-dimethylpropyl)-2-[4-(hydroxymethyl)-1-piperidyl]-4-methoxy-N-(6-methyl-2-pyridyl)benzamide (0.3 g, 0.7 mmol) in dichloromethane (7 mL) and stir at room temperature for 60 min. Remove all volatiles and purify the crude material by filtration through a silica plug eluting with 1% EtOAc/hexanes to 50% EtOAc/hexanes to give the title compound (0.550 g, 81%). ES/MS m/z 424 (m+H).

Preparation 7

N-(2,2-dimethylpropyl)-2-(4-ethynyl-1-piperidyl)-4-methoxy-N-(6-methyl-2-pyridyl)benzamide

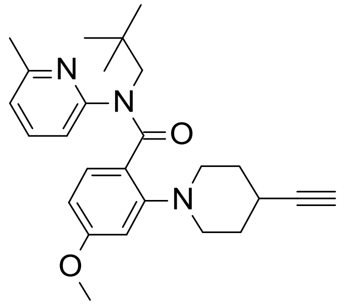

Add Dimethyl-1-diazo-2-oxopropylphosphonate (0.12 g, 0.62 mmol) N-(2,2-dimethylpropyl)-2-(4-formyl-1-piperidyl)-4-methoxy-N-(6-methyl-2-pyridyl)benzamide (0.22 g, 0.52 mmol) in methanol (8.7 mL) at room temperature and stir for 4 hr. Remove the volatiles. Add ethyl acetate, wash with an aqueous solution (5%) of $NaHCO_3$ (60 mL) and dry over Na_2SO_4, filter and remove the solvent in vacuo. Purify the crude material by filtration through a silica plug eluting with 1% EtOAc/hexanes TO 50% EtOAc/hexanes to give the title compound (0.22 g, 96%). ES/MS m/z 420 (m+H).

Preparation 8

methyl (3S)-3-cyclopropyl-3-(3-hydroxy-4-iodo-phenyl)propanoate

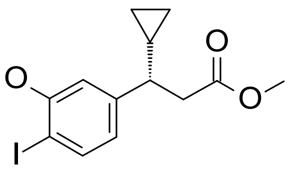

Prepared as in PCT WO 2014/130608

Preparation 9

methyl (3S)-3-cyclopropyl-3-[2-[1-[2-[2,2-dimethylpropyl-(6-methyl-2-pyridyl)carbamoyl]-5-methoxy-phenyl]-4-piperidyl]benzofuran-6-yl]propanoate

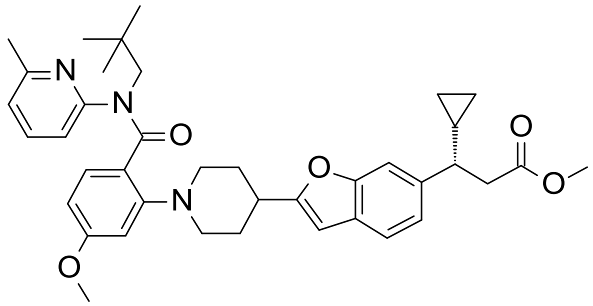

Add copper(I) iodide (0.009 g, 0.047 mmol), Bis (triphenyl phosphine)palladium(II) dichloride (16 mg, 0.023 mmol), triethyl amine (0.97 mL, 0.47 mmol) to methyl (3S)-3-cyclopropyl-3-(3-hydroxy-4-iodo-phenyl)propanoate (0.165 g, 0.47 mmol) in acetonitrile (2 mL). Stir the place on a preheated heating block at 80°C for 2 min. Add N-(2,2-dimethylpropyl)-2-(4-ethynyl-1-piperidyl)-4-methoxy-N-(6-methyl-2-pyridyl)benzamide (0.2g, 0.48 mmol) in 10 mL of acetonitrile and continue to stir at 80°C for 90 min. Dilute the reaction with ethyl acetate, pour the mixture over sat. ammonium chloride and separate the layers. Dry the organic layer with magnesium sulfate. Separate by filtration and concentrate the solution to remove volatiles. Purify the residue by silica gel chromatography eluting with a gradient of 100% hexane to 40% EtOAc:hexanes to give title compound (0.12 g, 39%) as a colorless oil. ES/MS m/z 638 (m+1).

Preparation 10

Methyl (2S,3R)-3-(3-aminophenyl)-3-cyclopropyl-2-methyl-propanoate

Compound **1**

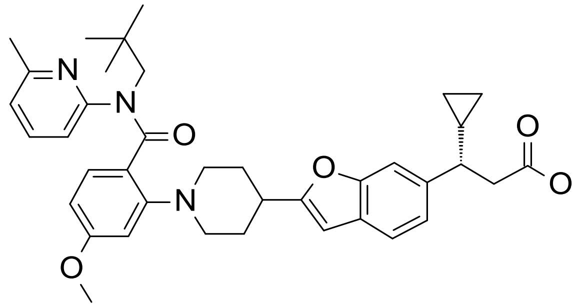

Add methyl (3S)-3-cyclopropyl-3-[2-[1-[2-[2,2-dimethylpropyl-(6-methyl-2-pyridyl)carbamoyl]-5-methoxy-phenyl]-4-piperidyl]benzofuran-6-yl]propanoate (0. 120 g, 0.18 mmol) to THF (6 mL), MeOH (6 mL), and water (6 mL). Add lithium hydroxide monohydrate (0.078 g, 1.85 mmol). Stir the mixture at 60°C overnight. Cool the reaction mixture at room temperature and concentrate to dryness. Treat the residue with ice water (10 mL) and adjust the pH to 4-5 with 0.1 N HCl solution. Extract the suspension with EtOAc (100 mL). Wash the organic extract with brine (25 mL), dry over Na_2SO_4, filter the solution, and concentrate to dryness. Purify the residue by silica gel flash chromatography, eluting with a gradient of 0 to 70% EtOAc in hexanes to give of the title compound (0.110 g, 91 %). ES/MS m/z 624 (m-H). 1HNMR (399.80 MHz, d6-DMSO) δ 12.05 (bs, 1H), 7.43 (d, J= 8.0 Hz, 1H), 7.38 (s, 1H), 7.29-7.27 (m, 1H), 7.15-7.12 (m, 1H), 7.10-7.07 (m, 1H), 6.84-6.81 (m, 1H), 6.53-6.51 (m, 2H), 6.45-6.44 (m, 1H), 6.24-6.22 (m, 1H), 4.19-4.18 (m, 1H), 4.06-4.04 (m, 1H), 3.67 (s, 4H), 2.79-2.78 (m, 5H), 2.34-2.33 (m, 5H), 1.99-1.97 (m, 4H), 1.66-1.65 (m, 1H), 1.10-1.08 (m, 1H), 0.72 (s, 10H), 0.53-0.52 (m, 1H), 0.32-0.30 (m, 2H), 0.18-0.17 (m, 1H).

Supplementary Reference:

1 Hauge, M. *et al.* GPR40 (FFAR1) - Combined Gs and Gq signaling in vitro is associated with robust incretin secretagogue action ex vivo and in vivo. *Mol Metab* **4**, 3-14, doi:10.1016/j.molmet.2014.10.002 (2015).
